# Therapeutic Effect of Hydrogen Sulfide-Releasing L-Dopa Derivative ACS84 on 6-OHDA-Induced Parkinson’s Disease Rat Model

**DOI:** 10.1371/journal.pone.0060200

**Published:** 2013-04-03

**Authors:** Li Xie, Li-Fang Hu, Xing Qi Teo, Chi Xin Tiong, Valerio Tazzari, Anna Sparatore, Piero Del Soldato, Gavin Stewart Dawe, Jin-Song Bian

**Affiliations:** 1 Department of Pharmacology, Yong Loo Lin School of Medicine, National University of Singapore, Singapore, Singapore; 2 Institute of Neuroscience & Department of Pharmacology, Soochow University, Suzhou, Jiangsu, China; 3 Dipartimento di Scienze Farmaceutiche, Università degli Studi di Milano, Milan, Italy; 4 CTG Pharma, Milan, Italy; University of Bologna & Italian Institute of Technology, Italy

## Abstract

Parkinson’s disease (PD), characterized by loss of dopaminergic neurons in the substantia nigra, is a neurodegenerative disorder of central nervous system. The present study was designed to investigate the therapeutic effect of ACS84, a hydrogen sulfide-releasing-L-Dopa derivative compound, in a 6-hydroxydopamine (6-OHDA)-induced PD model. ACS84 protected the SH-SY5Y cells against 6-OHDA-induced cell injury and oxidative stress. The protective effect resulted from stimulation of Nrf-2 nuclear translocation and promotion of anti-oxidant enzymes expression. In the 6-OHDA-induced PD rat model, intragastric administration of ACS84 relieved the movement dysfunction of the model animals. Immunofluorescence staining and High-performance liquid chromatography analysis showed that ACS84 alleviated the loss of tyrosine-hydroxylase positive neurons in the substantia nigra and the declined dopamine concentration in the injured striatums of the 6-OHDA-induced PD model. Moreover, ACS84 reversed the elevated malondialdehyde level and the decreased glutathione level *in vivo*. In conclusion, ACS84 may prevent neurodegeneration via the anti-oxidative mechanism and has potential therapeutic values for Parkinson’s disease.

## Introduction

Parkinson’s disease is an age-related progressive degenerative disorder, which is associated with the loss of dopaminergic neurons in the substantia nigra (SN) and leads to motor disorder like bradykinesia, resting tremor, rigidity, and postural instability [1]–[3]. Mitochondria dysfunction and oxidative stress are believed to play an important role in the pathogenesis of PD [3]. To date, Levodopa (L-Dopa) treatment is the most effective medication for Pakinson’s disease as it compensates for the dopamine deficiency [4]. However, L-Dopa does not arrest the progression of PD and long term treatment induces side effects like dyskinesia [5]–[7] and accelerates the neuron degeneration due to oxidative stress [8]–[11].

Hydrogen sulphide (H_2_S), an endogenous gasotransmitter, has been recognized to have crucial physiological functions in central nervous system. Reports have suggested that H_2_S is involved in introducing long-term potentiation (LTP) [12], [13], regulating calcium homeostasis [14], [15] and suppressing oxidative stress [16], [17]. Besides the physiology functions, H_2_S also plays important roles in pathological processes of neurodegenerative diseases. Our group has demonstrated that H_2_S is able to attenuate neuroinflammation induced by lipopolysaccharide [18] and amyloid-β [19], suppress oxidative stress induced by hydrogen peroxide [20], and protect cells against cell injury induced by neurotoxins such as rotenone [21] and 6-OHDA [22]. We and other groups also found that intraperitoneal injection of NaHS (an H_2_S donor) [23] or inhalation of H_2_S [24] asserted protective effects against Parkinson’s disease animal models.

**Figure 1:**
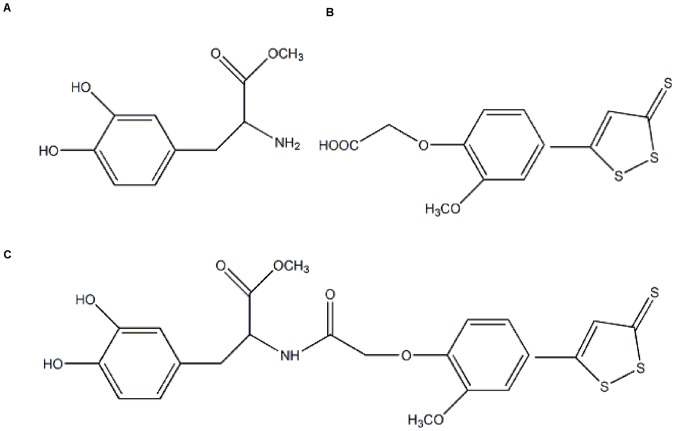
Chemical structure of L-Dopa, ACS50 and ACS84. The chemical structures of (A) L-Dopa methyl ester, (B) ACS50, and (C) ACS84 are displayed. ACS84 is a hybrid of L-Dopa methyl ester and ACS50. The dithiole-thione group in ACS50 is believed to release H_2_S in cells.

Based on these reports, it was speculated that the combination of L-Dopa and H_2_S may have a potential therapeutic value [25], [26]. ACS84, as shown in Fig. 1, is a hybrid compound derived from L-Dopa methyl ester (Fig. 1A) and ACS50 (a H_2_S-releasing moiety) (Fig. 1B), which can penetrate blood brain barrier and release H_2_S in cells [25]. Although the effect of ACS84 on PD is not known yet, ACS84 and other H_2_S-releasing L-Dopa derivatives have been proved to suppress neuroinflammation and inflammation-induced cell injury, and elevate glutathione level while inhibit monoamine oxidase B activity [25]. Further investigation also suggested that ACS84 protected cells against amyloid β-induced cell injury via attenuation of inflammation and preservation of mitochondrial function [27].

6-OHDA is a widely accepted experimental toxin for induction of PD model, which selectively kills dopaminergic neurons [28]. Sharing similar structure with dopamine, it can be uptaken by dopaminergic neurons through dopamine reuptake transporters. 6-OHDA generates reactive oxygen species (ROS) in the cells and finally induces oxidative stress and cell injury [29]. In this study, we used both *in vitro* and *in vivo* models of 6-OHDA to evaluate the therapeutic potential of ACS84 in PD treatment.

## Materials and Methods

The experimental protocol was approved by the Institutional Animal Care and Use Committee (IACUC) of National University of Singapore. All animal works were carried out strictly in accordance with IACUC regulations.

### Chemicals and Reagents

All chemicals, antibodies for detecting tyrosine hydroxylase and LDH assay kit were purchased from Sigma (Sigma, St. Louis, MO). Antibodies for detecting Nrf-2 were purchased from Santa Cruz Biotechnology (Santa Cruz, CA). The Glutathione Assay Kit, TBARS Assay Kit and Superoxide Dismutase Assay Kit were purchased from Cayman Chemical (Ann Arbor, Michigan). ACS84 was prepared as previously described [25].

### Cell Culture and Treatment

The human neuroblastoma cell line, SH-SY5Y, was obtained from the American Type Culture Collection (Manassas, VA, USA). Cells were maintained in Dulbecco’s modified Eagle’s Medium (DMEM) supplemented with 10% foetal bovine serum (FBS) and 0.05 U·mL-1 penicillin and 0.05 mg/ml streptomycin at 37°C in a humidified atmosphere containing 5% CO_2_/95% air. Cells were plated onto 96-well plates for viability tests and ROS generation assay, or 35 mm dishes and incubated overnight. Regular medium was replaced with low-serum medium (0.5% FBS/DMEM) before treatment. For Nrf-2 translocation, medium was changed to non-serum medium and incubated for another 12 h before treatment with ACS84, L-Dopa or NaHS for 1–8 h.

### Cell Viability Assay

Cell viability was measured using the MTT reduction assay as described previously [21]. At the end of each treatment, MTT was added to each well at a final concentration of 0.5 mg·mL^−1^ and the cells were further incubated at 37°C for 4 h. The insoluble formazan was dissolved in dimethyl sulphoxide. Colorimetric determination of MTT reduction was measured at 570 nm with a reference wavelength of 630 nm.

### Lactate Dehydrogenase (LDH) Release Assay

At the end of treatment, cell culture medium was collected and briefly centrifuged. The supernatants were transferred into wells in 96-well plates. Equal amounts of lactate dehydrogenase assay substrate, enzyme and dye solution were mixed. A half volume of the above mixture was added to one volume of medium supernatant. After incubation at room temperature for 30 min, the reaction was terminated by the addition of 1/10 volume of 1N HCl to each well. Spectrophotometrical absorbance was measured at a wavelength of 490 nm and reference wavelength of 690 nm.

### Reactive Oxygen Species (ROS) Measurement

Formation of reactive oxygen species (ROS) was evaluated using non-fluorescent dye 2′, 7′- dichlorofluorescin diacetate (DCFH-DA), which freely penetrates cells and yields the highly fluorescent product dichlorofluorescein (DCF) by ROS oxidation. Following ACS84, L-Dopa or NaHS treatment, cells were rinsed with PBS solution and incubated with Hank’s Buffered Salt Solution (HBSS) containing DCFH-DA dye (10 µM final concentration) 30 minutes in the dark. 6-OHDA was added then and fluorescence was read immediately for 1 h, at an excitation wavelength (Ex) of 490 nm and an emission wavelength (Em) of 520 nm.

### Superoxide Dismutase (SOD) Activity Determination

SOD activity was measured in cells using the Cayman Chemical Superoxide Dismutase Assay Kit (Cayman Chemicals, Inc, Ann Arbor, MI). Briefly, cells were sonicated in 20 mM HEPES buffer, pH 7.2, containing 1 mM EGTA, 210 mM mannitol and 70 mM sucrose, on ice. After centrifugation, the supernatant was collected. Reaction was initiated by adding diluted xanthine oxidase to all wells, and then the plate was incubated on a shaker at room temperature for 20 min. The absorbance was read at 450 nm.

### Reverse Transcription-PCR

The mRNA levels of GclGclM, HO-1 and β-Actin were determined by two-step reverse transcription PCR. In brief, total RNA was extracted using TRIzol® reagent (Invitrogen, Carlsbad, CA, USA). Homogenized samples were then incubated at room temperature for 5 min. Chloroform was added and tubes were shaked vigorously by hand for 15 min followed by incubation for 3 min at room temperature once more. Samples were centrifuged at 12000 g for 15 min at 4°C. Colourless upper aqueous phase was transferred to a new tube containing isopropanol and incubated for 10 min at 25°C followed by centrifugation at 12000 g for 10 min at 4°C. Supernatant was thrown away and RNA pellet was washed with 70% ethanol. RNA concentration was determined with NanoDrop Spectrophotometer (ND-1000, NanoDrop Technology). Equal amounts of RNA samples obtained were reverse transcribed into cDNA using iScriptTM cDNA synthesis kit (Bio-Rad). Reverse transcription was performed at 25°C (for 5 min), 42°C (for 30 min) and 85°C (for 5 min). The resulting cDNAs were PCR-amplified using Taq DNA polymerase kit (i-DNA Biotechnology). The specific PCR primer sequences used were as follows: GclC (forward primer 5′-TGAGATTTAAGCCCCCTCCT-3′ and reverse primer 5′- TTGGGATCAGTCCAGGAAAC-3′) [NM_001498.3], and GclM (forward primer 5′- TTTGGTCAGGGAGTTTCCAG-3′and reverse primer 5′-ACACAGCAGGAGGCA AGATT-3′) [NM_002061.2] [30]; HO-1 (forward primer 5′-CAGGCA GAGAATGCTGAGTTC-3′ and reverse primer 5′- GCTTCACATAGCGCTGCA-3′) [NM_002133.2] [31]; and β-actin forward primer (5′-AAGAGAGG CATCCTCACCCT-3′) and β-actin reverse primer (5′-TACATGGCTGGGG TGTTGAA-3′) [NM_001101.3] [32]. PCR conditions were set as 95^o^C (for 30 sec), 58^o^C (for 30 sec), and 72^o^C (for 30 sec) for 30 cycles. PCR products were separated on a 1% agarose gel and stained with ethidium bromide. The optical densities of the mRNA bands were analyzed with GelDoc-It Imaging Systems.

### Western Blot

For Western blot analysis, the cells were washed with ice-cold PBS and homogenized with lysis buffer containing 150 mM NaCl, 25 mM Tris (pH7.5), 5 mM EDTA, 1% Nonidet P-40, (additional 10 mM NaF and 1 mM Na3VO4 were immediately added before detection of phosphorylation) and protease inhibitor cocktail tablet (Roche Diagnostics, Penzberg, Germany). The lysates were then vigorously shaken on ice for one hour and centrifuged at 13,200 g at 4°C for 10 min. After that, the supernatant was collected and denatured by SDS-sample buffer. Epitopes were exposed by boiling the protein samples at 100°C for 5 min. The protein samples were separated by SDS-PAGE gel and subsequently transferred onto the nitrocellulose membrane (Whatman). Membranes were then blocked with 10% milk/TBST buffer for one hour and incubated with appropriate primary antibodies at 4°C overnight. On the second day, membranes were washed and incubated with appropriate HRP-conjugated second antibody. Visualization was performed using ECL® (plus/advanced chemiluminescence) kit (GE healthcare, UK). The density of the bands on film was quantified by Image J software (National Institute of Health, USA).

### Nuclear and Cytoplasmic Protein Fractionation

The preparation of cytoplasmic and nuclear extracts was performed using the Nuclear Extract kit (Active Motif) according to manufacturer’s instruction. Briefly, cells were scraped using cell lifter in ice-cold PBS. Cell pellet obtained after centrifugation was re-suspended in a hypertonic buffer and incubated on ice for 10 min. After the addition of detergent, the suspension was centrifuged. The supernatant (cytoplasmic fraction) was collected. The remaining nuclear pellet was re-suspended in complete lysis buffer. After vortex and centrifugation, the supernatant (nuclear fraction) was collected.

### 6-OHDA Induced PD Rat Model

Male Sprague-Dawley (SD) rats (180–220 g) were anesthetized with ketamine (75 mg/kg, i.p.) and xylazine (10 mg/kg, i.p.). After that, the rats were placed in a stereotaxic apparatus (Stoelting Instruments, Wood Dale, IL, USA). 6-OHDA (8 µg 6-OHDA hydrobromide dissolved in 4 µl sterile saline containing 0.02% ascorbic acid) was unilaterally injected into the left striatum (coordinates from bregma: AP, +1.0 mm; ML, +3.0 mm; DV, −4.5 mm) with a Hamilton syringe (0.46 mm in diameter, blunt tip) at a rate of 0.5 µl per minute. The needle was left in place for 3 min and then slowly withdrawn in the subsequent two to three minutes. Sham-operated rats were injected with 4 µl saline containing 0.02% ascorbic acid into the left striatum and served as controls in this study. After surgery, the rats were kept in cages and exposed to a 12∶12 h light-dark cycle with unrestricted access to tap water and food.

### Behavioural Test

Three weeks after surgery, the animals’ tendency to rotate in response to apomorphine (0.5 mg/kg, s.c.) was tested. This test was re-performed one week later, i.e. four weeks after surgery. Only those rats consistently showing at least 7 turns per min in both tests were considered as the successfully induced PD-like model. These PD-like rats were then divided into different groups receiving different treatments, vehicle- or ACS84- administered group. In addition, the sham-operated rats also received vehicle treatment. These treatments continued for another 3 weeks. The rotational behaviour was monitored at one week interval till the end of treatment, and the behaviour tests were conducted before drug treatments in order to avoid disturbance. After 3 weeks of treatment, the rats were euthanized by CO_2_ and brain tissues were collected for assays.

### Immunofluorescence Staining

The immunofluorescence staining was performed according to the procedures as previously described with some modification [23]. At the end of behavioural test, the animals were anesthetized and perfused with sterile saline and subsequently with 4% paraformaldehyde (PFA). After that, rats were decapitated. The brain were then collected and immersed into 4%PFA for postfix at 4°C overnight. These brain samples were transferred into 15% sucrose in phosphate buffered saline (PBS) overnight at 4°C and subsequently to 30% sucrose solution till the brain sunk to the tube bottom. Thereafter, the brain were sectioned on a cryostat at a thickness of 30 µm and mounted onto the poly-l-lysine coated slides. These sections were stored at −70°C for further experimentation.

The sections were permeabilized with 0.3% Triton X-100/PBS for 10 min and blocked with 10% BSA in PBS for another 30 min. After that, the sections were incubated with mouse monoclonal anti-TH antibody (1∶500, Sigma, St. Louis, MO, USA) for 2 h at room temperature and followed with appropriate goat anti-mouse secondary antibody incubation for one hour.

### Lipid Peroxidation Assessment

The level of MDA, a marker of oxidative stress, was measured using TRABS assay kit (Cayman Chemical). The assay was performed according to the manufacturer’s instructions. In brief, brain tissues were lyzed with chilled RIPA buffer and sonicated for 15 s at 40 V over ice. After centrifugation at 1600 g for 10 min at 4 ^o^C, the supernatant was collected for further analysis. The MDATBA adduct formed by the reaction of MDA in samples and TBA supplied in the assay kit under high temperature (100 ^o^C) and acidic conditions. Reaction product was measured colorimetrically at 540 nm with a spectrophotometer (Tecan M200). The content of MDA in samples expressed as micromolar of MDA produced per gram of protein.

### Concentration Determination of Dopamine and its Metabolites

High-performance liquid chromatography (HPLC) was used to detect concentration of dopamine and its metabolites in the brain tissues. The method was described in the previous report [33]. The striatum was sonicated in 0.1 M perchloric acid. Homogenates were centrifuged at 14,000 *g* for 20min at 4251658240°C. The supernatants were collected and adjusted the pH value around 3. After that, the supernatants were subjected to HPLC (HTEC-500; Eicom, Kyoto, Japan) equipped with the column (EICOMPAK SC-3ODS; Eicom, Kyoto, Japan) and electrochemical detector (AD Instruments Pty Ltd., Castle Hill, NSW, Australia). Data was analyzed using PowerChrom (eDAQ, Australia).

### Statistical Analysis

Statistical significance was assessed with one-way analysis of variance (ANOVA) followed by a post hoc (Bonferroni) test for multiple group comparison for using PASW 18 (IBM, NY). For two group comparison, student T-test was used. Differences with p value less than 0.05 were considered statistically significant.

## Results

### Protective Effect of ACS84 on 6-OHDA-induced Cell Injury

**Figure 2:**
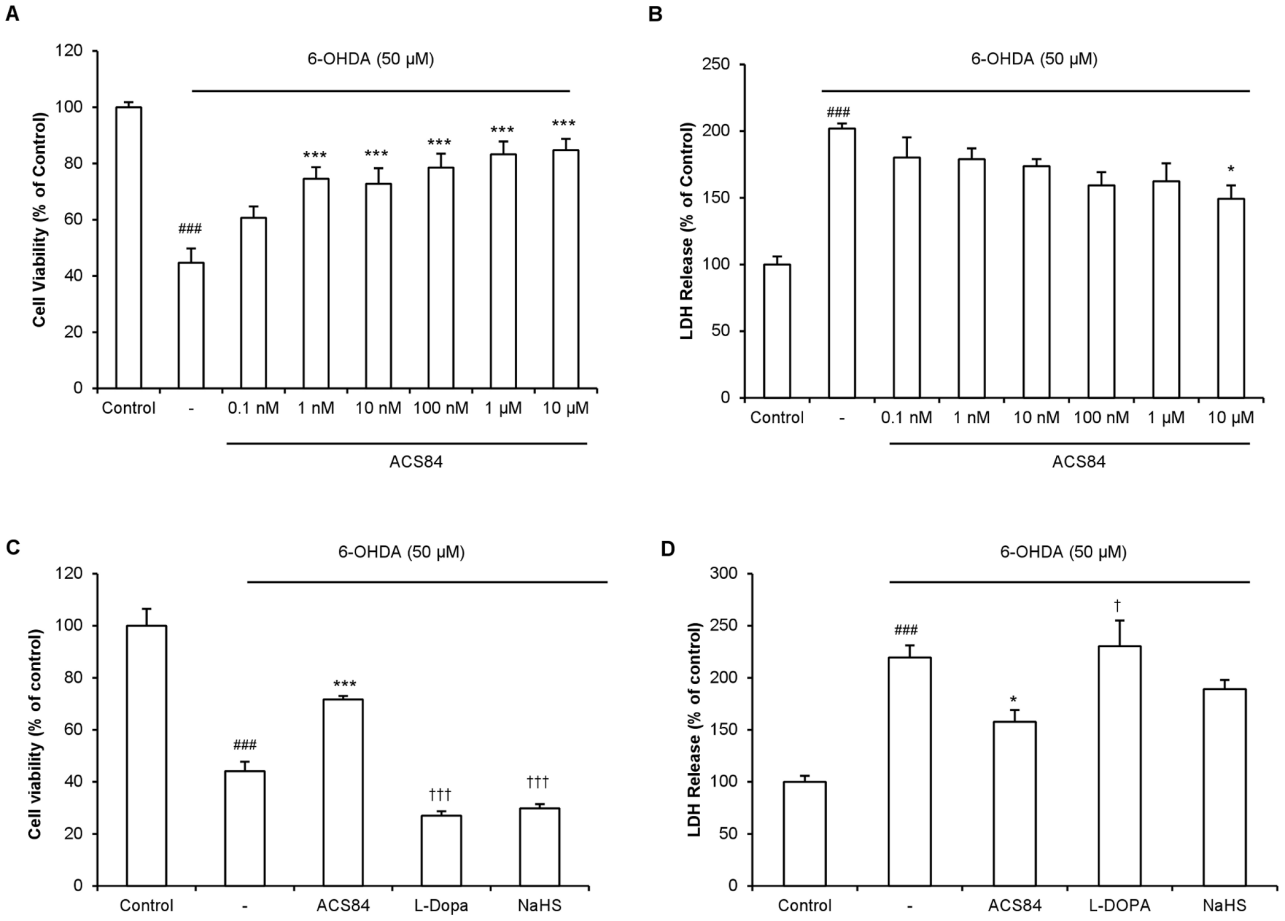
Protective effect of ACS84 against cell injury induced by 6-OHDA in SH-SY5Y cells. (A–B): Dose dependent effects of ACS84 on (A) cell viability and (B) LDH release in the 6-OHDA-treated (50 µM) SH-SY5Y cells. Cells were pretreated with ACS84 at different concentrations for 1 h before the addition of 6-OHDA. The results were obtained at 12 h (MTT assay) or 6 h (LDH release assay) after the treatment with 6-OHDA. (C–D): Effect of ACS84, L-Dopa and NaHS at 10 µM on cell viability (C) and LDH release (D) in SH-SY5Y cells treated with 6-OHDA. Data are presented as mean ± SEM, n = 5–9, ^###^ *P*<0.001 versus control; *P<0.05, ***P<0.001 versus 6-OHDA-treated cells; ^†^ *P*<0.05, ^†††^ *P*<0.001 versus ACS84-treated cells.

To evaluate the protective effect of ACS84 against 6-OHDA-induced cell injury in SH-SY5Y cells, cells were pretreated with ACS84 at different concentrations for 1 h before the treatment of 6-OHDA (50 µM) for another 6 h or 12 h. As shown in Fig. 2A and 2B, ACS84 at 0.1 nM to 10 µM concentration-dependently increased cell viability (Fig. 2A) and decreased LDH release (Fig. 2B) in cells treated with 6-OHDA. As ACS84 is a compound constituted by L-Dopa and H_2_S-releasing moiety, we examined whether L-Dopa or H_2_S alone would be able to produce similar protective effect as ACS84 did. As shown in Fig. 2C and 2D, neither L-Dopa nor NaHS (an H_2_S donor) at the equal molar concentration (10 µM) was sufficient to exert the similar protective effects against 6-OHDA-induced cell injury as ACS84 did (Fig. 2C and 2D). This is consistent with our previous findings that NaHS produced significant protective effects only when its concentration is higher than 100 µM [22]. These data suggest that ACS84 may produce stronger protective effects than either L-Dopa or NaHS alone.

### ACS84 Reduced the Oxidative Stress Induced by 6-OHDA

**Figure 3:**
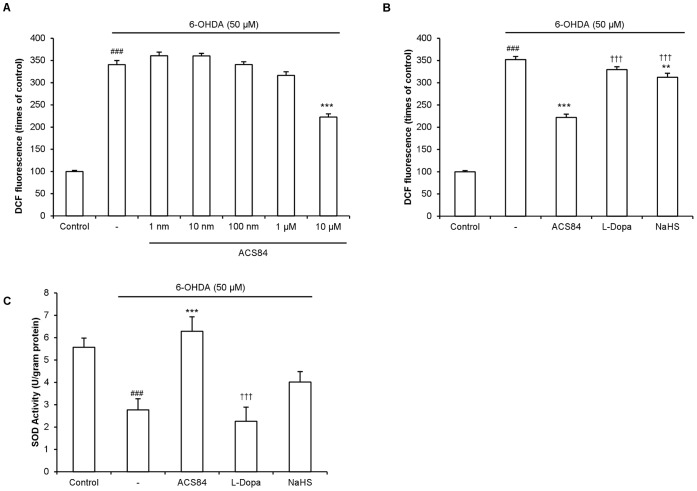
Effect of ACS84 on oxidative stress induced by 6-OHDA in SH-SY5Y cells. (A) Dose dependent effect of ACS84 on ROS generation in the 6-OHDA-treated (50 µM) SH-SY5Y cells. Cells were pretreated with ACS84 at different concentrations for 4 h. DCFDAH_2_ (10 µM) was given 30 min before the addition of 6-OHDA (50 µM). The results were obtained after the treatment with 6-OHDA for 1 h. (B–C) Effect of ACS84, L-Dopa and NaHS at 10 µM on ROS generation (B) and SOD (C) in SH-SY5Y cells treated with 6-OHDA. SOD activity was measured 4 h after 6-OHDA treatment. Data are presented as mean ± SEM, n = 4–8, ^###^ *P*<0.001 versus control; *P<0.05, **P<0.01, ***P<0.001, versus 6-OHDA-treated cells; ^†††^ *P*<0.001, versus ACS84-treated cells.

As it was well-accepted that 6-OHDA selectively killed dopaminergic neuron via generating reactive oxygen species (ROS) and inducing oxidative stress in the cells, we proceeded to examine the effect of ACS84 on 6-OHDA-induced ROS formation in SH-SY5Y cells. As shown in Fig. 3A, ACS84 at 10 µM significantly reduced ROS production induced by 6-OHDA (50 µM). Although NaHS also suppressed the 6-OHDA-induced ROS formation, this effect was much weaker when compared with that caused by ACS84 (Fig. 3B). Superoxide dismutases (SODs) are a family of enzymes which catalyze the dismutation of superoxide and play important roles in cell homeostasis. As shown in Figure 3C, ACS84, but not L-Dopa and NaHS, at 10 µM completely abolished the inhibitory effect of 6-OHDA on SOD activity.

### ACS84 Promoted Anti-oxidative Stress Associated Gene Expression

**Figure 4:**
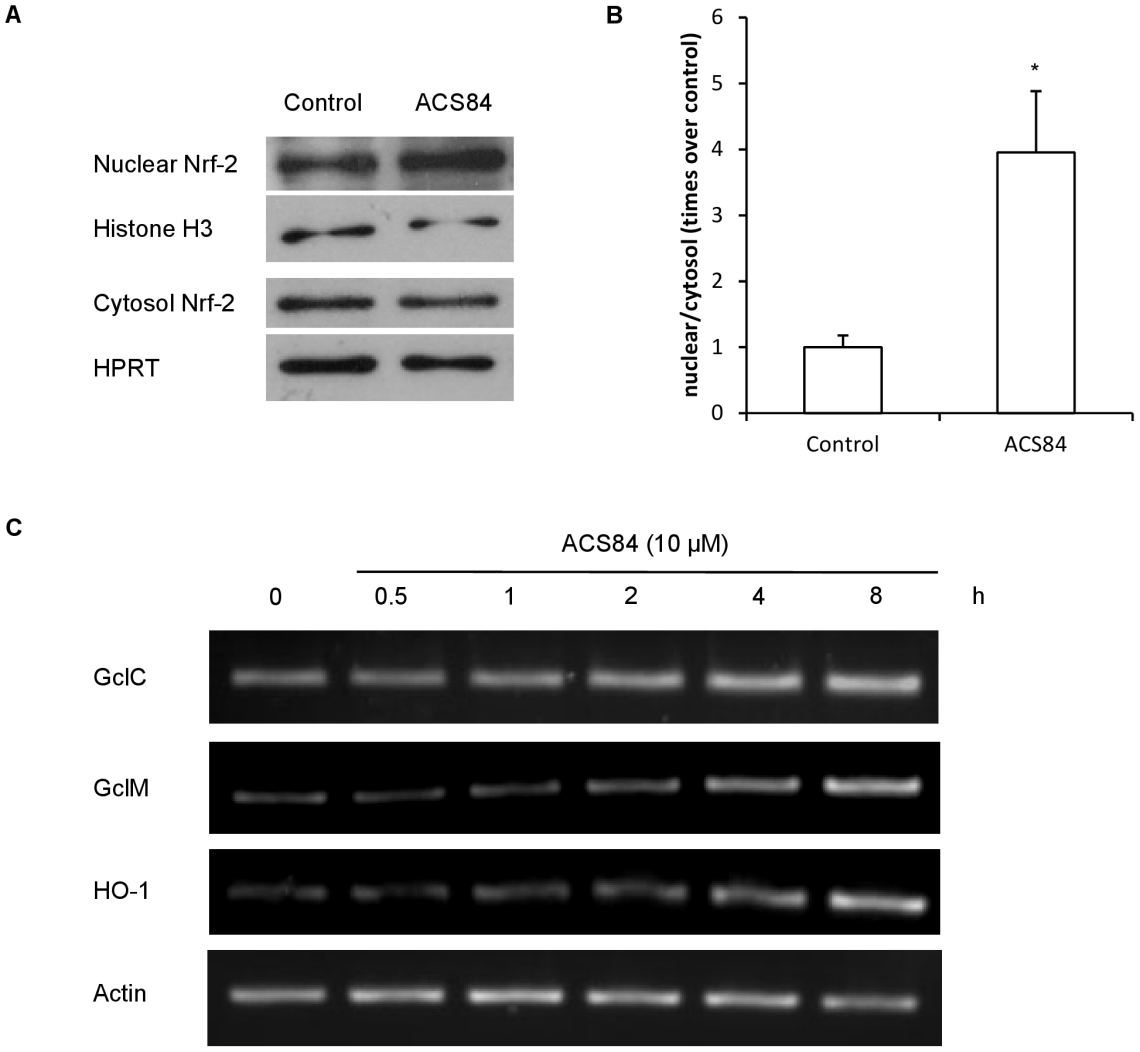
Effects of ACS84 on the expression and translocation of antioxidant enzymes in SH-SY5Y cells. (A) Western blotting analysis showing that treatment with ACS84 for 4 h promoted the nuclear accumulation of Nrf-2 in SH-SY5Y cells. Densitometric analysis performed by normalizing nuclear Nrf-2 to cytosol Nrf-2 signals. Data were expressed as mean ± SEM, *P<0.05, n = 5 (B) RT-PCR showing that ACS84 treatment induced the mRNA expression of GclC, GclM and HO-1 after 4 h. Samples were collected from three independent experiments.

Cells express anti-oxidant enzymes to protect against oxidative stress and most of these enzyme-coding genes contain anti-oxidant reaction element (ARE). NF-E2-related factor 2 (Nrf-2) is an important transcription factor which binds to ARE and initiates the expression of anti-oxidant enzymes, including glutamate cysteine ligase (GCL) and heme oxidase-1 (HO-1). Western blotting analysis shows that ACS84 treatment for 4 h promoted the nuclear translocation of Nrf-2 from cytosol to nuclear (Fig. 4A). Glutamate cysteine ligase catalytic subunit (GclC), Glutamate cysteine ligase modifier subunit (GclM) and HO-1 are three important Nrf-2 target genes. RT-PCR also showed that the mRNA levels of these three genes were significantly elevated after treatment with ACS84 for 4 h (Fig. 4B). These data suggested that ACS84 induced Nrf-2 nuclear translocation and promoted the expression of anti-oxidant enzymes, which contributed to the protection against 6-OHDA-induced oxidative stress.

### ACS84 Ameliorated Behaviour Symptom in the Unilateral 6-OHDA Rat Model

**Figure 5:**
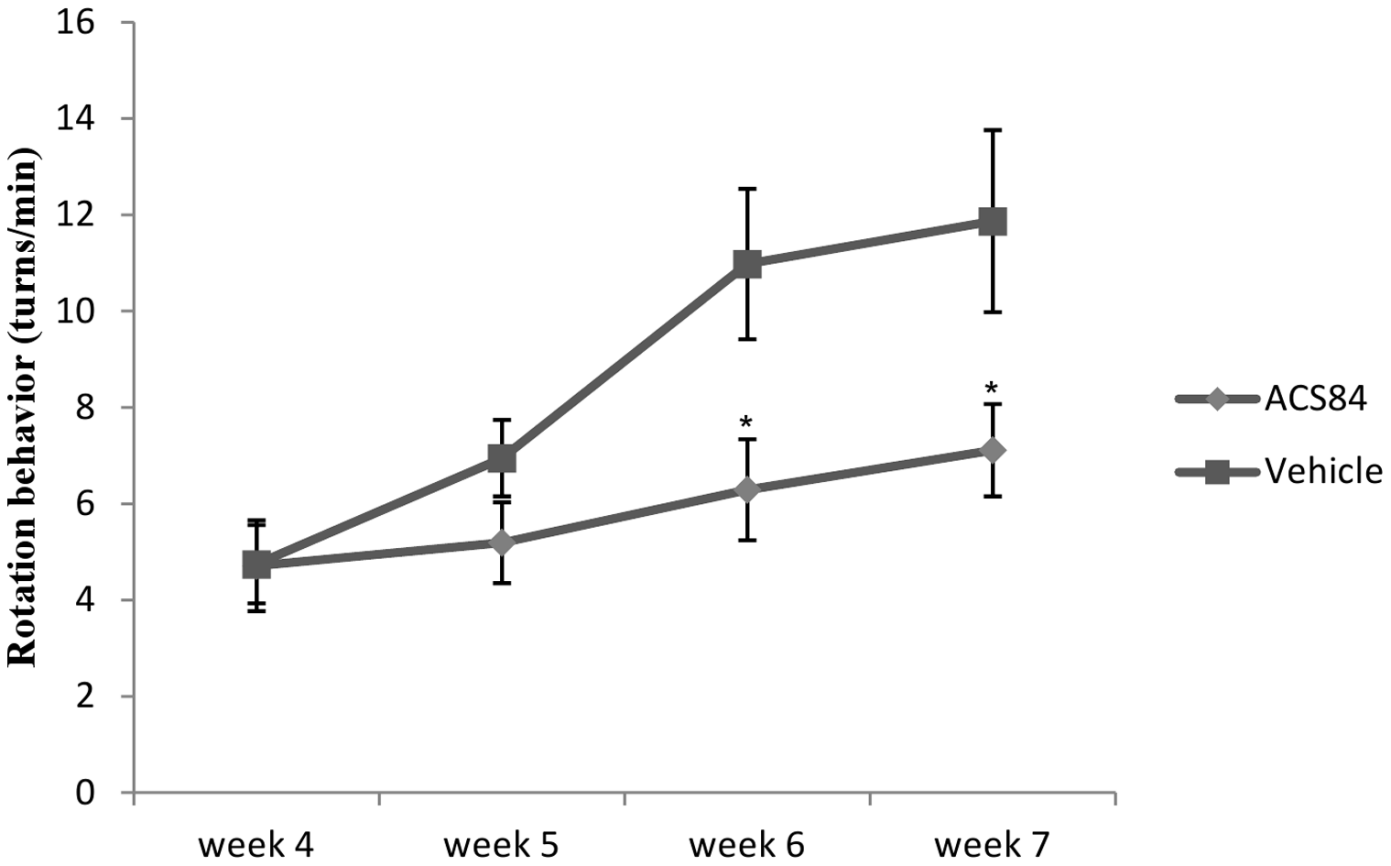
Treatment with ACS84 ameliorated the rotational behaviour in the unilateral 6-OHDA-lesioned rats. ACS84 (10 mg kg^−1^ day^−1^, i.g) was given daily from the 5th to 7th week after 6-OHDA lesion. Data are presented as mean ± SEM, n = 9. *P<0.05 versus the values in Vehicle group at the corresponding time point.

To evaluate the therapeutic effect of ACS84 on Parkinson’s disease, we established the unilateral 6-OHDA lesion rat model. Four weeks after 6-OHDA lesion, the PD rats were injected intragastrically with vehicle or ACS84 (10 mg/kg) daily and the treatment continued for 3 weeks. As shown in Fig. 5, ACS84 significantly ameliorated the rotation behaviour after 2 weeks of treatment, which indicated that the administration of ACS84 may alleviate the behaviour disorder in Parkinson’s disease.

### ACS84 Attenuated the Degeneration of Dopaminergic Neuronal in SN

**Figure 6:**
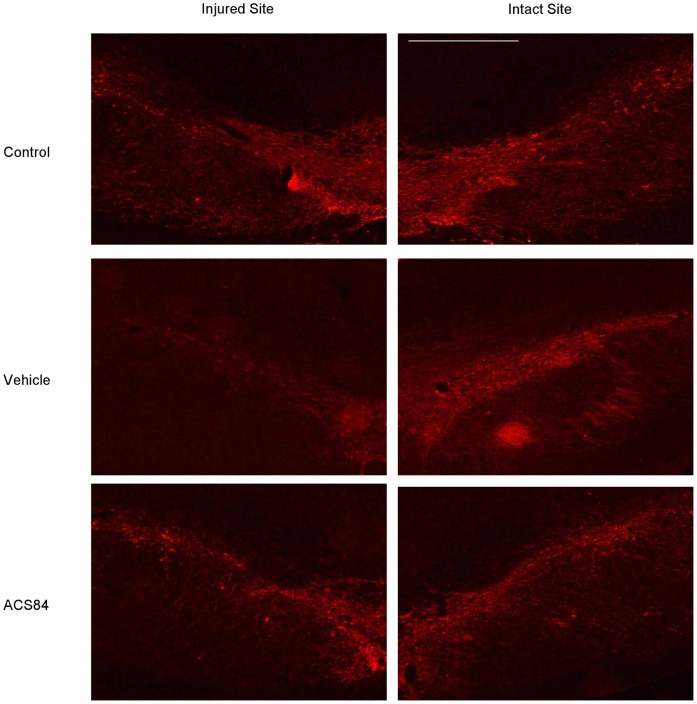
Effect of ACS84 on 6-OHDA-induced TH+ neuronal degeneration. Immunofluorescence staining showed that ACS84 (10 mg kg^−1^ day^−1^, i.g) alleviated TH+ neuron loss in SN of 6-OHDA-lesioned PD rats. Photos were taken at x100 magnification, and the white bar indicated 0.1 µm. Samples were collected from two independent experiments.

The movement dysfunction of the PD model is mainly associated with the loss of dopaminergic neurons in the SN and striatum. From the immunostaining results (Fig. 6), unilateral 6-OHDA lesion destroyed most of the tyrosine hydroxylase positive (TH+) neurons in SN pars compacta in the injured hemisphere, while the administration of ACS84 remarkably attenuated the effects. As tyrosine hydroxylase is the rate-limiting enzyme in dopamine synthesis, this data suggests that ACS84 may preserve the function of dopaminergic neurons in 6-OHDA-injured.

### ACS84 Relieved the Declined Dopamine Level in the 6-OHDA-injured Striatum

We further examined the dopamine and its metabolic products levels in the injured striatum. The concentrations of dopamine and the dopamine metabolites, dihydroxyphenylacetic acid (DOPAC) and homovanillic acid (HVA) were measured with HPLC. As shown in Table 1, 6-OHDA lesion significantly decreased the concentrations of dopamine, DOPAC and HVA in the injured striatum, while ACS84 treatment reversed these effects. These data were comparable with the results of behaviour test and immunofluorescence staining, indicating that ACS84 efficiently alleviated the loss of dopaminergic neurons and the deficient of dopamine in the striatum. In our experiments, we observed a significant increase of dopamine/HVA ratio but not dopamine/DOPAC ratio in ACS84 animals compared with vehicle group, indicating that ACS84 might also suppress catechol-*O*-methyl transferase (COMT) activity.

**Table 1:** Effect of ACS84 on dopamine and its metabolites in 6-OHDA-lesioned striatum.

Treatment	Dopamine	DOPAC	HVA	Dopamine/DOPAC	Dopamine/HVA
Sham	8.25±1.01	2.54±0.71	1.47±0.23	4.90±0.74	5.96±0.46
Vehicle	1.61±0.45#	1.00±0.24	0.71±0.10	2.03±0.72#	2.24±0.50#
ACS84	7.35±1.62*	3.81±0.89*	2.15±0.41*	2.15±0.41	4.90±0.67*

The concentration of dopamine and its metabolites in 6-OHDA-lesioned striatum were measured using HPLC. Units for Dopamine, DOPAC and HVA concentrations were ng/g tissue. Data are presented as mean ± SEM, n = 6–8.

# p<0.05 versus Sham group and *p<0.05 versus Vehicle group.

### ACS84 Suppressed the Oxidative Stress in the Injured Striatum

**Figure 7:**
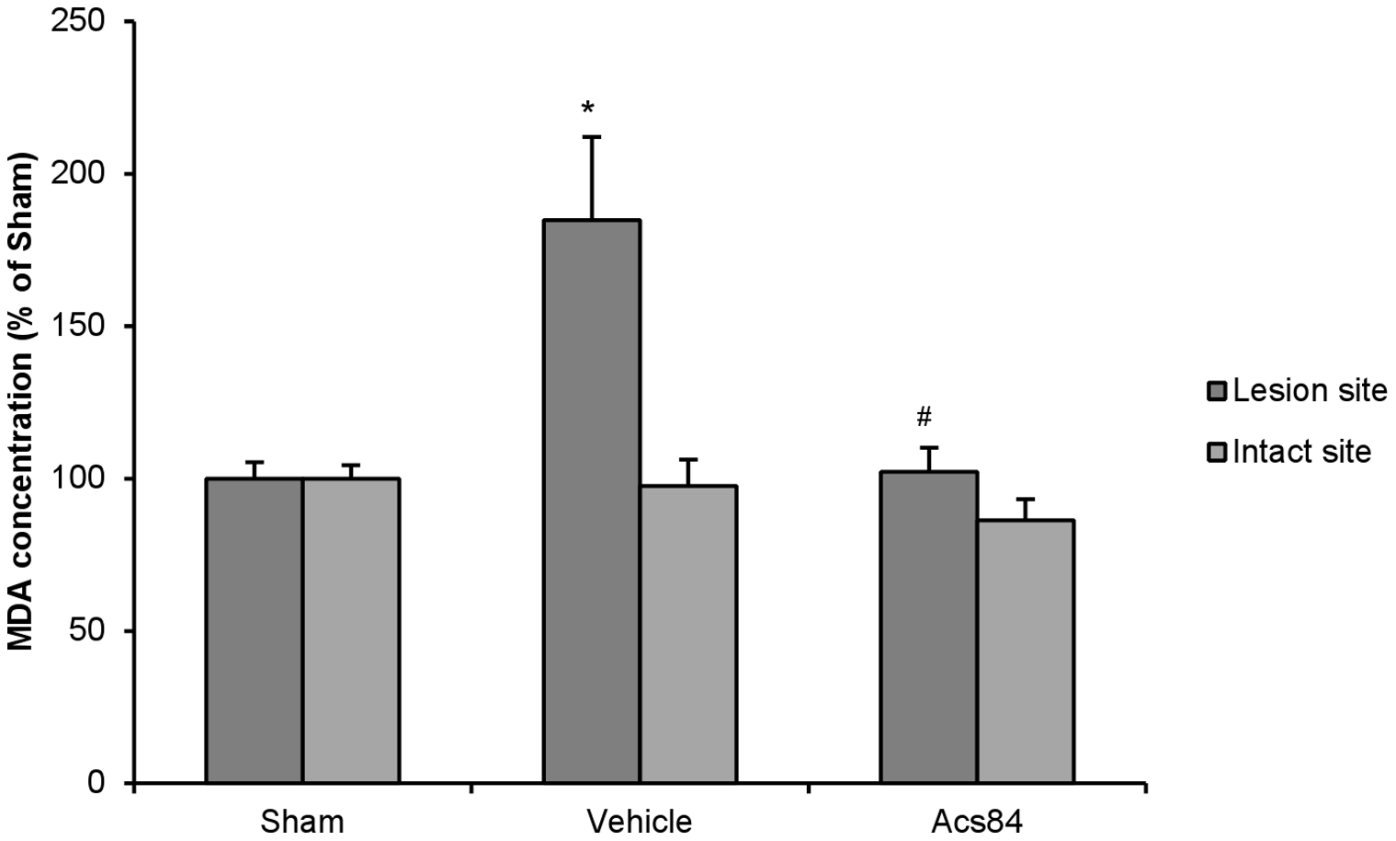
Effect of ACS84 on oxidative stress in the striatum of unilateral 6-OHDA-lesioned PD rat model. ACS84 treatment (10 mg kg^−1^ day^−1^, i.g) alleviated the increased MDA production. Data are presented as mean ± SEM, n = 4–6. *P<0.05 versus lesion site of Sham group and ^#^ *P*<0.05 versus lesion site of Vehicle group.

Malondialdehyde (MDA) is a marker for lipid peroxidation to indicate the oxidative stress level in the striatum. As shown in Fig. 7, 6-OHDA induced the elevation of MDA production in the injured striatum, when compared to sham and healthy striatum. ACS84 treatment significantly suppressed this effect. This data suggested that ACS84 protected dopaminergic neurons degeneration by suppressing oxidative stress in the brain.

## Discussion

The symptoms of Parkinson’s disease are associated with the loss of dopaminergic neurons and the deficiency of dopamine in the SN and striatum, and oxidative stress plays a crucial role in the pathology of neurodegeneration [3], [34]. Though the traditional L-Dopa treatment for PD patients could compensate for the dopamine deficiency and alleviate the behaviour disorder, long-term usage of L-Dopa has its disadvantages and has been proven to enhance oxidative stress [8]–[11].

H_2_S has been recognized as an anti-oxidant [20], [35], [36] and our group has demonstrated the protective effect of H_2_S in 6-OHDA and rotenone-induced PD models [21]. ACS84 is a hybrid compound which is derived from L-Dopa and one H_2_S-releasing moiety, ACS50 [25]. ACS84 and other H_2_S-releasing L-Dopa derivatives have been shown to have therapeutic potential as they suppressed microglia activation [25]. In the present study, we used 6-OHDA-induced PD model to investigate the therapeutic effect of ACS84.

It is interesting to find that ACS84 showed significant protective effect against 6-OHDA-induced cell injury and oxidative stress in SH-SY5Y cells, while at equal molar concentration of both L-Dopa and NaHS failed to achieve. Though it has been reported that NaHS was able to protect the cells against apoptosis and oxidative stress, our results suggested that ACS84 showed a better therapeutic potential as it produced protective effect at a lower dose, at which concentration NaHS failed to protect neuronal cells. We postulated that the better effect of ACS84 may be due to its slower H_2_S-releasing rate. In addition, ACS84 releases H_2_S intracellularly by mitochondria [26]. This may further enhance the action efficiency of endogenous H_2_S. Another possibility is that the metabolites of ACS84 may interact with endogenous H_2_S or its generating enzyme, cystathionine β-synthase (CBS) to produce stronger effect than exogenous application of NaHS. More experiments are warranted to investigate the exact underlying mechanism.

Gcl and HO-1 are anti-oxidant enzymes involving in the cellular stress defence system. Both coding gene contain ARE *cis*-element. When activated, transcript factor Nrf-2 translocates from the cytoplasm to the nuclear and binds to the ARE. This initiates the gene expression of anti-oxidant enzymes [37]–[40]. Our results showed that ACS84 treatment induced nuclear translocation of Nrf-2 and promoted the gene transcription of GclC, GclM and HO-1, which further indicated that ACS84 may attenuate oxidative stress via stimulating Nrf-2/ARE pathway to increase anti-oxidant enzymes in the cells.

S-sulfhydration of cysteine residues in proteins has now been recognized as one of the key mechanisms for H_2_S physiology [41]–[45]. Tao et al proposed that cysteine residues may serve as H_2_S “receptors” in cells and H_2_S may attack disulfide bonds in proteins to regulate protein functions [46]. A recently published report indicated that H_2_S S-sulfhydrated Keap1 at cysteine-151, which enhanced the release of Nrf-2 from Keap-1 and upregulated Nrf-2 activity [47]. This report was on a par with our results and suggested that S-sulfhydration may be a potential mechanism of ACS84 in cells.

We used unilateral 6-OHDA PD rat model to evaluate the protective effect of ACS84 *in vivo*. This model is mainly designed to study the oxidative injury in PD pathogenesis process [29]. Our group has demonstrated that the protective effects of H_2_S involved suppression of NADPH oxidase in this model [21]. In the present study, we also found that post-treatment of ACS84 preserved TH+ neurons in SN, and maintained the dopamine levels and relieved the movement dysfunction. These data suggest that ACS84 is of potential therapeutic value for Parkinson’s disease. We believe that the suppression of oxidative stress was the main mechanism in the protective effect of ACS84. We have proven that ACS84 was efficient in reducing ROS formation and was able to stimulate the expression of anti-oxidant enzymes *in vitro*. Along with these results obtained from SH-SY5Y cells, we also found that ACS84 treatment reversed the effect of 6-OHDA on MDA levels. Having such an effect on the two oxidative stress indicators, these data proved that ACS84 plays a role as an anti-oxidant in the dopaminergic neurons. Although we found that ACS84 produced significant anti-oxidant effect, unfortunately, we failed to find ACS84 produced better effect against movement dysfunction in 6-OHDA-induced PD model when compared with L-dopa (data not shown). This result implies that ACS84 is not more efficacious to treat PD symptoms than L-Dopa alone in our observation period (7 weeks). Longer period observation and other animal models may be needed in future to determine which one, ACS84 or L-dopa, produces longer and better effects.

It was reported that ACS84 intravenous injection increased the dopamine level in the rat brain [25]. Our results also indicated that ACS84 elevated dopamine levels in the 6-OHDA-injured striatum. ACS84 may increase dopamine in two aspects: firstly, ACS84 may release L-Dopa which would be further catalysed into dopamine by DOPA decarboxylase; secondly, ACS84 protected dopaminergic neuron degeneration, thus alleviated dopamine deficient.

It was reported that ACS84 and other similar H_2_S-releasing compounds would inhibit the monoamine oxidase B activity in neonatal rat striatal astroglial cell primary cultures and SH-SY5Y cells. [25], [48] We also got similar results from primary cultured astroglial cells (data not shown). However, from our HPLC results, we did not observe the significant decline of DOPAC/dopamine ratio in the striatum homogenates from ACS84-treated rats. These data may suggest that the inhibitory effects of ACS84 on monoamine oxidase B activity were not as efficient under physiological conditions as in cell cultures. In contrast, ACS84 significantly decreased the dopamine/HVA ratios, suggesting that ACS84 might suppress COMT activity, which was responsible for the dopamine-HVA and DOPAC-HVA turnover in dopaminergic neurons.

As we found ACS84 efficacy requires a chronic administration regimen in this study, there might be some transcriptional and translational alterations in neurons. It was found that ACS84 attenuated the down-regulated protein expression of tyrosine hydrolase (TH) in our PD model. In addition, the anti-oxidation-related genes were also upregulated in cells treated with ACS84 through Nrf-2 pathway. Our data suggest that the effects of ACS84 may result from translational alternations, despite that the initial process of S-sulfhydration itself is reversible.

In conclusion, we have demonstrated the neuroprotective effect of ACS84, one H_2_S-releasing L-Dopa derivative, in the 6-OHDA models of Parkinson’s disease. ACS84 suppressed 6-OHDA-induced cell injury and ROS generation and induced anti-oxidant enzymes expression via Nrf-2 stimulation. Moreover, ACS84 also ameliorated the movement dysfunction and dopaminergic neuron degeneration in unilateral 6-OHDA PD rat model by suppressing oxidative injury. Our results imply that ACS84 has the potential to be developed to a new drug to treat Parkinson’s disease. However, toxic effects of ACS84 also need to be determined before any conclusion is drawn.
